# Unannounced Meals in the Artificial Pancreas: Detection Using Continuous Glucose Monitoring

**DOI:** 10.3390/s18030884

**Published:** 2018-03-16

**Authors:** Charrise M. Ramkissoon, Pau Herrero, Jorge Bondia, Josep Vehi

**Affiliations:** 1 Institut d’Informàtica i Aplicacions, Universitat de Girona, Campus de Montilivi, s/n, Edifici P4, 17071 Girona, Spain; charrise.ramkissoon@udg.edu; 2 Centre for Bio-Inspired Technology, Institute of Biomedical Engineering, Imperial College London, London SW7 2AZ, UK; pherrero@imperial.ac.uk; 3 Instituto Universitario de Automática e Informática Industrial, Universitat Politènica de València, Camino de Vera, s/n, 46022 València, Spain; jbondia@isa.upv.es; 4 Centro de Investigación Biomédica en Red de Diabetes y Enfermadades Metabólicas Asociadas (CIBERDEM), Instituto de Salud Carlos III, 28028 Madrid, Spain

**Keywords:** meal detection, artificial pancreas, unannounced meals, type 1 diabetes, physiological models, Unscented Kalman Filter

## Abstract

The artificial pancreas (AP) system is designed to regulate blood glucose in subjects with type 1 diabetes using a continuous glucose monitor informed controller that adjusts insulin infusion via an insulin pump. However, current AP developments are mainly hybrid closed-loop systems that include feed-forward actions triggered by the announcement of meals or exercise. The first step to fully closing the loop in the AP requires removing meal announcement, which is currently the most effective way to alleviate postprandial hyperglycemia due to the delay in insulin action. Here, a novel approach to meal detection in the AP is presented using a sliding window and computing the normalized cross-covariance between measured glucose and the forward difference of a disturbance term, estimated from an augmented minimal model using an Unscented Kalman Filter. Three different tunings were applied to the same meal detection algorithm: (1) a high sensitivity tuning, (2) a trade-off tuning that has a high amount of meals detected and a low amount of false positives (FP), and (3) a low FP tuning. For the three tunings sensitivities 99 ± 2%, 93 ± 5%, and 47 ± 12% were achieved, respectively. A sensitivity analysis was also performed and found that higher carbohydrate quantities and faster rates of glucose appearance result in favorable meal detection outcomes.

## 1. Introduction

Closed-loop systems intended for blood glucose (BG) control in people with type 1 diabetes (T1D), i.e. artificial pancreas (AP) systems, are still dependent on feed-forward actions such as meal announcements to achieve effective control. However, many studies have reported that a high number of missed meal boluses occur, especially in adolescents during insulin pump therapy. Several studies have reported a link between glycated hemoglobin (HbA1c ) levels and missed meal boluses [1,2,3] showing an average increase in HbA1c of 4 mmol/mol (0.3%) during a 2-week period due to missed meal boluses [1]. These findings have been further compounded by Patton et al. [4] who found a higher correlation between poor HbA1c levels and missed meal boluses compared to frequency of daily BG measures. Increases in HbA1c lead to an increased risk of long-term complications such as retinopathy, nephropathy, neuropathy, heart disease, and stroke. It is predicted that this missed-meal-bolus behavior will carry over to AP therapy and, therefore, a means to reduce poor outcomes due to unannounced meals must be implemented.

An AP system regulates BG levels using a continuous glucose monitor (CGM) informed controller that adjusts insulin infusion via an insulin pump. In healthy subjects, hypo- and hyperglycemia are counteracted by a physiological control system that includes pancreatic hormones such as insulin, glucagon, and amylin [5]. In T1D, this control system is lacking, which is especially evident after a meal when a large postprandial increase in BG is experienced. Meals are difficult for the AP to mitigate due to the delayed onset of current rapid-acting insulin formulations and the 5–15 min time lag in the interstitial space experienced by CGM readings [6,7,8].

A few studies have used unannounced meals simply as a challenge to see how well their controller works when a premeal bolus is not given [9,10] and have found that their controller is able to overcome small unannounced meals but have difficulty with larger meals [10]. Larger unannounced meals therefore require meal detection systems and strategies to combat postprandial hyperglycemia.

Dassau et al. [11] designed a meal detection system that uses binary detection that is triggered by a voting scheme. This system was trained with a MiniMed CGMS Gold dataset and tested in clinic using Freestyle Navigator CGM readings. Lee and Bequette [12] detected a meal based on certain logical conditions of first and second derivatives of glucose levels, and then a finite impulse response filter was applied to estimate the meal size. Then, Lee et al. [13] refined the logical conditions and generated a series of meal impulses, a moderately sized bolus was then given according to the meal impulses. Cameron et al. [14] developed a probabilistic detection algorithm based on a set of meal shapes with the meal start time and postprandial glucose appearance estimated at the same time. Later on, Cameron and Niemeyer [15] came up with a multi-model method to detect and estimate unannounced meals. The method is also probabilistic based but assumes a constant meal absorption shape. Chen et al. [16] and Weimer et al. [17] are able to detect meals utilizing a bin-counting heuristic that counts the number of decisions generated using a dual parameter-invariant statistics leveraged from a linearized physiological model. Xie and Wang [18] use a multi-model approach to detect meals, meal start time, and meals size using a Variable State Dimension method, which uses a switching criteria to switch between models with different state dimensions. Turksoy et al. [19] propose a method for meal detection and bolus estimation based on an estimated rate of glucose appearance (Ra). Finally, Mahmoudi et al. [20] use an adaptive Unscented Kalman Filter (UKF) and two redundant CGM sensors to detect meals based on glucose predicted by a model and CGM values. A meal is detected when CGM values of both sensors fall outside the 95% confidence interval of the predicted model.

Lee et al. [13] and Cameron et al. [14] have achieved remarkable in silico results in an adult cohort in terms of postprandial hyperglycemia mitigation with a time in range (70–180 mg/dL) of 90% and 89%, respectively. However, these results have not been translated into a clinical setting where detection modules are still unable to prevent a large majority of postprandial hyperglycemia and in turn result in many hypoglycemic events as seen in Turksoy et al. [21] and Cameron et al. [22]. The idea behind this work was to build a meal detection algorithm in the context of an AP system in a way that it can be used to trigger automatic feed-forward actions that safely and effectively mitigate postprandial hyperglycemia. Therefore, we hypothesize that the proposed approach to meal detection provides sufficient sensitivity and reliability to be effectively used within an AP system.

The main advantages of this work are: (1) the use of both meal size and composition as points of interest during the evaluation of the proposed algorithm and (2) the use of multiple tunings to assess algorithm usefulness when introducing meal mitigation actions.

## 2. Materials and Methods

**Figure 1:**
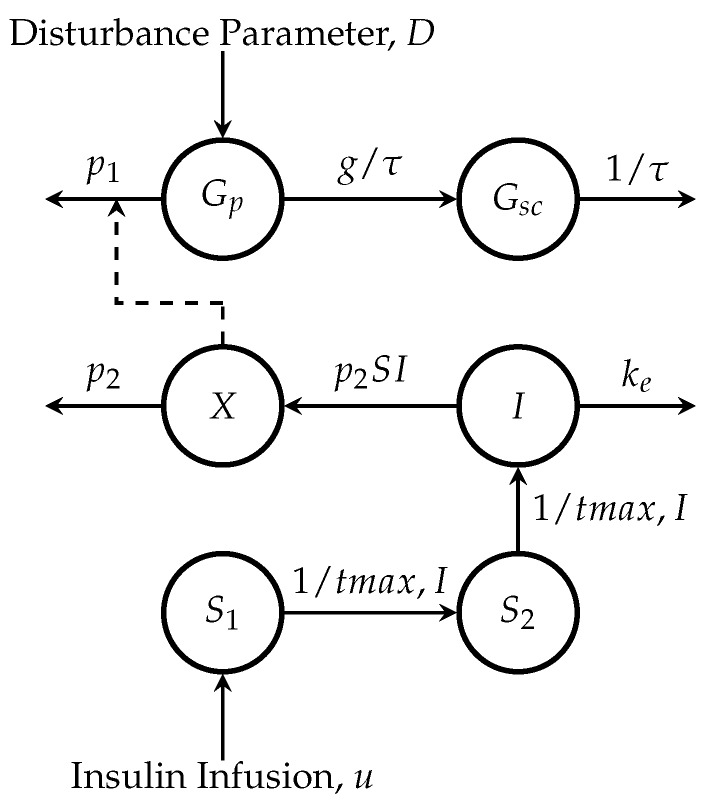
Compartmental model of glucose-insulin system. $G_{p}$ and $G_{sc}$ represent glucose concentrations in the accessible (plasma) and non-accessible (subcutaneous) compartments, *I* represents plasma insulin, and *X* represents a remote insulin compartment that accelerates glucose disappearance [23].

The meal detection algorithm presented in this paper was tuned and validated using a 14-day in silico simulation. A sensitivity analysis is also presented for meals over a 500-day period in ten adult subjects. The algorithm collects glucose and insulin infusion rate values and computes a disturbance parameter from an augmented minimal model (Figure 1) using an UKF. The cross-covariance between the glucose data and the forward difference of computed disturbance parameter is then calculated over three different sliding windows. A threshold is then applied, different for each sliding window. One threshold is applied to obtain a detection that has a high true positive (TP) value, the second is a trade-off tuning, which has both a high TP value and a low false positive (FP) value, and the third tuning has a very low FP value.

### 2.1. Minimal Model

A minimal model was employed for the use in state estimations for meal detection. The equations are provided in the following subsections.

#### 2.1.1. Glucose Subsystem

The model is comprised of the Bergman equations (see [23]):

(1) $$\frac{dG_{p}\left(t\right)}{dt}=-\left(p_{1}+X\left(t\right)\right)G_{p}\left(t\right)+p_{1}G_{b}+\frac{D(t)}{V_{G}},$$

(2) $$\frac{dX(t)}{dt}=-p_{2}X\left(t\right)+p_{2}S_{I}I\left(t\right),$$

where $G_{p}$ is plasma glucose concentration, *X* is proportional to insulin in the remote compartment, and $G_{b}$ is basal glucose. $p_{1}$ represents the rate at which glucose is removed from the plasma space independent of the influence of insulin. $V_{G}$ is the distribution volume, $p_{2}$ is the rate of disappearance of remote insulin from the remote insulin compartment, and $S_{I}=p_{3}/p_{2}$ is the insulin sensitivity. *D*, identified using an UKF (Equation (9)), is a lumped signal used to describe plasma glucose variations due to meals, exercise, and other various disturbances that are not described by the other parameters of the model.

The blood to interstitial glucose dynamics is described as a first-order linear system [24]:

(3) $$\frac{dG_{sc}\left(t\right)}{dt}=-\frac{1}{\tau }G_{sc}\left(t\right)+\frac{g}{\tau }G_{p}\left(t\right),$$

where $G_{sc}$ represents subcutaneous glucose, τ represents the time constant of the system and *g* is the static gain of the system.

#### 2.1.2. Insulin Subsystem

Insulin absorption [25] is described as:

(4) $$\frac{dS_{1}\left(t\right)}{dt}=u\left(t\right)-\frac{S_{1}\left(t\right)}{t_{max,I}},$$

(5) $$\frac{dS_{2}\left(t\right)}{dt}=\frac{S_{1}\left(t\right)-S_{2}\left(t\right)}{t_{max,I}}.$$

$S_{1}$ and $S_{2}$ are a two compartment chain representing the subcutaneous absorption of rapid-acting (e.g., Lispro) insulin and u(t) (μU/kg/min) represents administration (bolus and infusion) of insulin.

The plasma insulin concentration [25] is given by:

(6) $$\frac{dI(t)}{dt}=-k_{e}I\left(t\right)+\frac{1}{V_{I}}\cdot \frac{S_{2}\left(t\right)}{t_{max,I}},$$

where $k_{e}$ is the fractional elimination rate, $V_{I}$ is the distribution volume, and $t_{max,I}$ is the time-to-maximum insulin absorption.

The T1D subject model was discretised using a first forward difference derivative approximation (1 min step size) [26]. The mean population values found in Table 1 are utilized in this model.

### 2.2. Unscented Kalman Filter

The UKF proposed by [28] is a powerful technique used in nonlinear estimation and machine learning applications. Its foundation lies in the intuition that it is easier to approximate a probability distribution than it is to approximate an arbitrary nonlinear function or transformation [29]. Using the discretized model of Equations (1)–(6) and the inputs of glucose and insulin infusion rate obtained from a CGM and an insulin pump, respectively, a nonlinear state space model is derived:

(7) $$\begin{array}{c} x(k+1)=f(x(k),u(k))+w(k), \end{array}$$

(8) $$\begin{array}{c} y(k)=g(x(k))+v(k), \end{array}$$

where x(k) is the state vector, which has been augmented by D(t), with state equation:

(9) $$\frac{dD(k)}{dt}=0.$$

u(k) is the input and w(k) and v(k) are defined to be process and measurement noises, respectively. The nonlinear functions f(·) and g(·) are defined from Equations (1)–(6). The state estimations are used for meal detection as described in Section 2.3.

The nonlinear function, y=f(x) of variable *x* (dimension L) is applied to each point, in turn, to yield a cloud of transformed sigma points, $X_{i}$ in the form of matrix *X* (dimension 2L+1) according to the following:

(10) $$\begin{array}{cc} X_{0}\left(k-1\right) & =\hat{x}\left(k-1\right), \end{array}$$

(11) $$\begin{array}{ccc} X_{i}\left(k-1\right)=\hat{x}\left(k-1\right)+{\left(\sqrt[{}]{\left(L+\lambda )P(k-1\right)}\right)}_{i} & \; & i=1,\ldots ,L, \end{array}$$

(12) $$\begin{array}{ccc} X_{i}\left(k-1\right)=\hat{x}\left(k-1\right)-{\left(\sqrt[{}]{\left(L+\lambda )P(k-1\right)}\right)}_{i-L} & \; & i=L+1,\ldots ,2L, \end{array}$$

with scalar weights $W_{i}$ defined as:

(13) $$\begin{array}{c} W_{0}^{m}=\frac{\lambda }{L+\lambda }, \end{array}$$

(14) $$\begin{array}{c} W_{0}^{c}=\frac{\lambda }{L+\lambda }+\left(1-\alpha^{2}+\beta \right), \end{array}$$

(15) $$\begin{array}{ccc} W_{i}^{m}=W_{i}^{c}=\frac{1}{2(L+\lambda )} & \; & i=1,\ldots ,2L, \end{array}$$

where $\lambda =\alpha^{2}\left(L+\kappa \right)-L$ is a scaling parameter, α determines the spread of the sigma points around $\hat{x}$, κ is a secondary scaling parameter, and β is used to incorporate prior knowledge of the distribution of *x*. P(k−1) is the covariance matrix and ${\left(\sqrt[{}]{\left(L+\lambda )P(k-1\right)}\right)}_{i}$ is the *i*th column of the matrix square root, i.e., lower triangle Cholesky factorization.

The sigma vectors, $X_{i}\left(k-1\right)$ are then propagated through the nonlinear function, f(·) as follows:

(16) $$\begin{array}{ccc} X_{i}^{-}\left(k\right)=f[X_{i}\left(k-1\right),u\left(k\right)] & \; & i=0,\ldots ,2L. \end{array}$$

The statistics of the transformed points can then be calculated to form an estimate of the nonlinearly transformed mean ($\hat{x}$) and covariance (P(k)):

(17) $$\begin{array}{c} {\hat{x}}^{-}\left(k\right)=\sum_{i=0}^{2L}W_{i}^{m}{\hat{X}}_{i}^{-}\left(k-1\right), \end{array}$$

(18) $$\begin{array}{c} P^{-}\left(k\right)=\sum_{i=0}^{2L}W_{i}^{c}\left(X_{i}^{-}\left(k-1\right)-{\hat{x}}^{-}\left(k\right)\right){\left(X_{i}^{-}\left(k-1\right)-{\hat{x}}^{-}\left(k\right)\right)}^{T}+Q_{p}, \end{array}$$

where $Q_{p}$ is the covariance matrix of the process noise. The sigma points $X_{i}^{-}$ are propagated through the nonlinear function g(·) for the calculation of $Y_{i}^{-}$:

(19) $$\begin{array}{ccc} Y_{i}^{-}\left(k\right)=g[X_{i}^{-}\left(k\right),u\left(k\right)] & \; & i=0,\ldots ,2L. \end{array}$$

The measurement estimations are obtained from $X_{i}^{-}$ as:

(20) $$\begin{array}{c} {\hat{y}}^{-}\left(k\right)=\sum_{i=0}^{2L}W_{i}^{m}Y_{i}^{-}\left(k\right). \end{array}$$

The innovation and cross-covariance matrices are then calculated as:

(21) $$\begin{array}{c} P_{yy}\left(k\right)=\sum_{i=0}^{2L}W_{i}^{c}\left[Y_{i}^{-}\left(k\right)-{\hat{y}}^{-}\left(k\right)\right]{\left[Y_{i}^{-}\left(k\right)-{\hat{y}}^{-}\left(k\right)\right]}^{T}+Q_{m}, \end{array}$$

(22) $$\begin{array}{c} P_{xy}\left(k\right)=\sum_{i=0}^{2L}W_{i}^{c}\left[X_{i}^{-}\left(k\right)-{\hat{x}}^{-}\left(k\right)\right]{\left[Y_{i}^{-}\left(k\right)-{\hat{y}}^{-}\left(k\right)\right]}^{T}, \end{array}$$

where $Q_{m}$ represents the covariance of measurement noise. Lastly, the Kalman filter gain and the updated state vector estimation and covariance matrix are calculated:

(23) $$\begin{array}{c} K\left(k\right)=P_{xy}\left(k\right){\left(P_{yy}\left(k\right)\right)}^{-1}, \end{array}$$

(24) $$\begin{array}{c} \hat{x}\left(k\right)={\hat{x}}^{-}\left(k\right)+K\left(k\right)\left(y\left(k\right)-{\hat{y}}^{-}\left(k\right)\right), \end{array}$$

(25) $$\begin{array}{c} P\left(k\right)=P^{-}\left(k\right)-K\left(k\right)P_{yy}\left(k\right){\left(K\left(k\right)\right)}^{T}. \end{array}$$

The UKF requires the definition of initial conditions and tuning parameters, which in our case were selected as:

$$\begin{array}{cc} \hat{x}\left(0\right)= & \left[\begin{array}{c} G_{p}\left(0\right)\\ X(0)\\ I(0)\\ S_{1}\left(0\right)\\ S_{2}\left(0\right)\\ G_{sc}\left(0\right)\\ D(0) \end{array}\right]=\left[\begin{array}{c} BG(0)\\ 0\\ 0\\ u(0)\\ 0\\ BG(0)\\ 0 \end{array}\right],\\ & q=0.6103\;\;\;r=8.9614,\\ & Q_{p}=diag\left[q^{2}\;0\;0\;0\;0\;q^{2}\;q^{2}\right],\\ & P\left(0\right)=I_{7\times 7}\;\;\;Q_{m}=r^{2},\\ & \alpha =10^{-3}\;\;\;\kappa =0\;\;\;\beta =2, \end{array}$$

where $G_{sc}\left(0\right)$ is the first measured glucose value from a CGM, *q* is the standard deviation of the process noise, and *r* is the standard deviation of the measurement noise.

### 2.3. Meal Detection

After the states in Equations (1)–(6) are estimated using the UKF, the values of $G_{sc}\left(k\right)$ from the CGM simulated data and $D_{diff}\left(k\right)$, which is the forward difference of D(k), are scaled between −1 and 1 using minimum and maximum values determined a priori through simulation. Then, the biased estimate of the cross-covariance between the two sequences is calculated during windows of a specified length.

The true cross-covariance sequence of two jointly stationary random processes, $G_{sc_{n}}$ and $D_{diff_{n}}$, is the cross-correlation of mean-removed sequences [30],

(26) $$\Phi_{G_{sc},D}\left(m\right)=E\left\{\left(G_{sc}\left(n+m\right)-\mu_{G_{sc}}\right){\left(D_{diff}\left(n\right)-\mu_{D_{diff}}\right)}^{*})\right\},$$

where $\mu_{G_{sc}}$ and $\mu_{D_{diff}}$ are the mean values of the two stationary random processes and *E* is the expected value operator. The asterisk denotes complex conjugation.

The raw cross-covariances are computed as:

(27) $$c_{G_{sc},D_{diff}}\left(m\right)=\left\{\begin{array}{cc} \left(\sum_{n=0}^{N-m-1}\left(G_{sc}\left(n+m\right)-\frac{1}{N}\sum_{i=0}^{N-1}G_{sc}\left(i\right)\right)\left(D_{diff}^{*}\left(n\right)-\frac{1}{N}\sum_{i=0}^{N-1}D_{diff}^{*}\left(n\right)\right)\right)/N, & m\;\geq 0,\\ \left(c_{D_{diff},G_{sc}}^{*}\left(-m\right)\right)/N, & m\;<0, \end{array}\right.$$

where $G_{sc}\left(n\right)$ and $D_{diff}\left(n\right)$ are indexed from 0 to N−1, and $c_{G_{sc},D_{diff}}\left(m\right)$ from −(N−1) to N−1, where *N* is the number of samples. The raw cross-covariance are normalized by scaling by 1/N.

A meal is then detected as follows:

(28) $$Meal=\left\{\begin{array}{c} True,\;if\;c_{G_{sc},D_{diff}}\left(m\right)\geq threshold,\\ \;and\;D_{diff}\left(k\right)>0,\\ \;and\;G_{sc}\left(k\right)-G_{sc}\left(k-3\right)>0,\\ False,\;otherwise. \end{array}\right.$$

**Figure 2:**
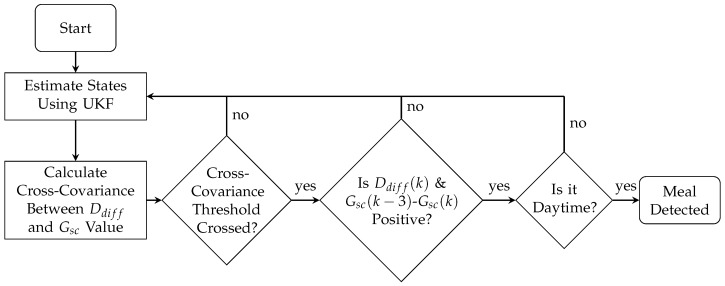
Flow chart of the meal detection algorithm. First, the states of a minimal model are estimated using an Unscented Kalman filter (UKF) and then the cross-covariance is found between the state estimation for the forward difference of the disturbance parameter ($D_{diff}$) and the glucose value $G_{sc}$ obtained from a continuous glucose monitor (CGM). A threshold is applied to the cross-covariance, and, once crossed, the last unscaled value of $D_{diff}$ and the slope with respect to the measurement 15 min ago (3 samples) of $G_{sc}$ are checked, if both are positive and it is daytime, a meal is detected.

**Figure 3:**
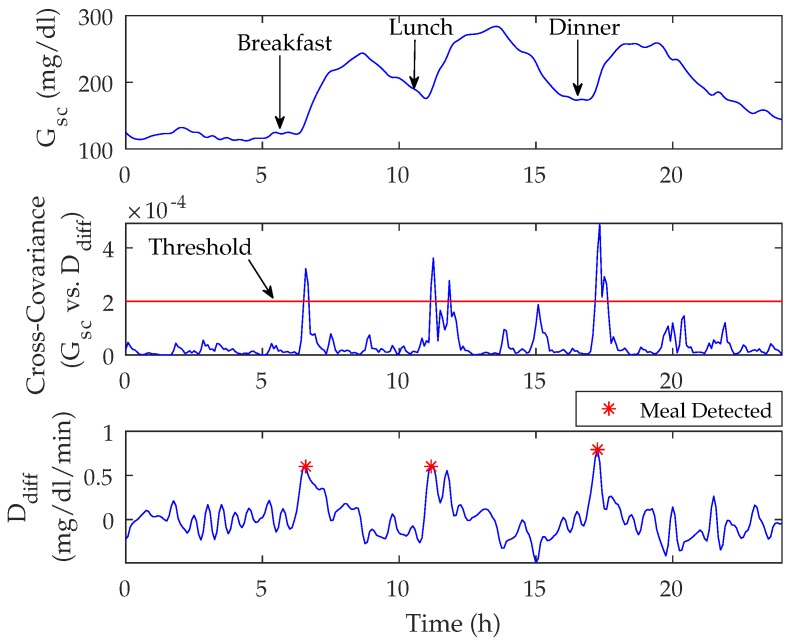
Illustration of the meal detection algorithm showing subcutaneous glucose ($G_{sc}$) values from a continuous glucose monitor values (top graph), cross-covariance (middle graph) and the forward difference of the disturbance parameter, $D_{diff}$ (bottom graph).

A meal was assumed to have been consumed if the cross-covariance between the $G_{sc}\left(n\right)$ and $D_{diff}\left(n\right)$ signals exceeded a pre-specified threshold and the last unscaled value of $D_{diff}$ and the slope of $G_{sc}$ with respect to the measurement 15 min ago (three samples) were positive (see Figure 2 and Figure 3). It should be noted that meals are not detected during the nighttime period (23 h–6 h) as a safety precaution.

### 2.4. Performance Metrics

Seven performance metrics were used: sensitivity, true positive (TP), false positive (FP), false negative (FN), false positive per day (FP/day), detection time, and Δ glucose. True negatives (TN) and specificity were not able to be determined in this framework due to the fact that one TP can span multiple time points depending on meal duration; however, a TN can be counted for each time sample that a negative occurrence is correctly identified. This skews the specificity to the higher end of the spectrum. Therefore, sensitivity and FP were used as measures of performance. Sensitivity measures the percentage of positive results that are correctly identified. Correct detection or a TP is when the algorithm has detected a meal from the beginning of the meal to 120 min after the time of the commencement of the meal. An FP is when detection is positive without a meal. Negative detection or an FN is when a meal has not been detected up to 120 min after ingestion. To compare the FP values between the two scenarios, the FP/day is used. Detection time reflects the time at which a TP is detected with respect to meal time and Δ glucose reflects the change in glucose from the start of the meal to the time when the meal was detected. All values are reported as mean ± standard deviation and median (5th%, 95th%).

### 2.5. Diabetes Simulation Scenario

The University of Virgina/Padova (UVA/Padova) Simulator [31] was used to build two different types of scenarios: (1) a meal detection tuning and validation scenario and (2) a meal detection sensitivity analysis scenario. Meal absorption rate and subcutaneous insulin absorption rate were varied at each meal according to a uniform distribution of ±10% and ±30%, respectively. Circadian insulin sensitivity variation (sinusoidal type with 24 h period) was implemented with random amplitude according to a uniform distribution of ±30% and random phase. Finally, CGM error was according to the default model available in the UVA/Padova simulator.

#### 2.5.1. Meal Detection Tuning and Validation Scenario

Two challenging 14-day scenarios were built: one scenario was used for tuning and the other was used for validation. These scenarios included: three meals per day with varying carbohydrate content and meal time following a normal distribution with mean 30 g at 8:30 h (breakfast), 60 grams at 13:00 h (lunch), and 50 g at 19:00 h (dinner). Coefficient of variance for the meal size was ±20%, and the standard deviation for meal time was ±10 min. For each meal, a meal absorption profile was selected randomly from a meal library of 11 meals, which are the meals of the 10 adults and the average adult provided by the UVA/Padova Simulator. There were 420 meals in total for all 10 subjects over the 14-day period.

#### 2.5.2. Meal Detection Sensitivity Analysis Scenario

A 500-day scenario with 10 adult subjects was built for a sensitivity analysis of the meal detection algorithm. Meals were uniformly distributed and ranged from 20 to 120 g of carbohydrates with meal time following a normal distribution with a standard deviation of ±10 min at 8:30 a.m. (breakfast), 1:00 p.m. (lunch), and 7:00 p.m. (dinner). For each meal, a meal absorption profile was selected randomly from a meal library of 49 meals. The meal library consisted of 31 fast absorption meals (over 60% of the ingested carbohydrates are absorbed within the first 2 h after meal time), three slow absorption meals (less than 80% of the ingested carbohydrates are absorbed within the first 4 h after meal time), and 15 medium absorption meals (otherwise). There were 15,000 meals in total for all 10 subjects over the 500-day period.

## 3. Results

### 3.1. UKF State Estimations

**Figure 4:**
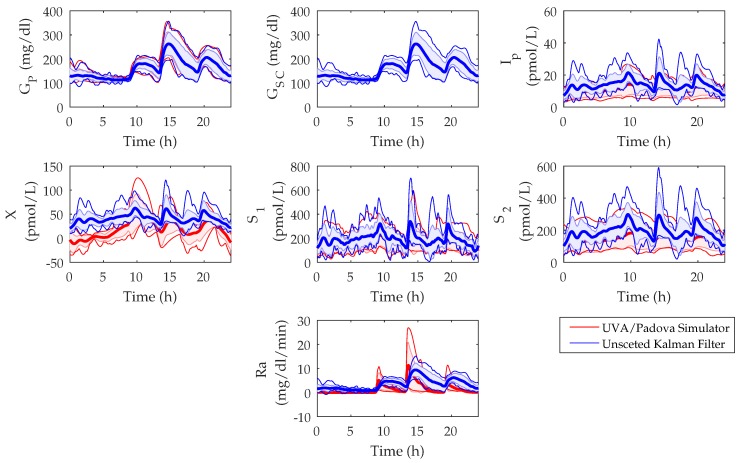
Population state estimations of the University of Virginia/Padova (UVA/Padova) Simulator versus the Unscented Kalman Filter over a 24 h period in silico. States shown are plasma glucose ($G_{P}$), subcutaneous glucose ($G_{SC}$), plasma insulin ($I_{p}$), remote insulin (*X*), insulin in the first subcutaneous compartment ($S_{1}$), insulin in the second subcutaneous compartment ($S_{2}$), and rate of glucose appearance (Ra) equated to the disturbance parameter, *D*. Graph reported as mean (bold lines) ± standard deviation (lightly shaded area), minimum (thin lower line), and maximum values (thin upper line).

The in silico state estimations of the UKF were compared to the model states of the UVA/Padova Simulator and the populational values are shown in Figure 4. The root mean squared error (RMSE) between the UVA/Padova Simulator and the UKF was calculated for each of the states over a 14-day period. The RMSE values of the mean populational values for $G_{p}$, $G_{sc}$, $I_{p}$, *X*, $S_{1}$, $S_{2}$, and Ra were found as 5.8 mg/dL, 0 mg/dL, 3.7 pmol/L, 25.6 pmol/L, 43.2 pmol/L, 60.1 pmol/L, and 2.8 mg/dL/min, respectively.

### 3.2. Meal Detection Algorithm

Three different tunings of the same detection algorithm were analyzed, one had the highest sensitivity, the second had a trade-off between the number of TP and FP, and the third had the lowest number of FP. These tunings were used to decipher the usefulness of the detection algorithms for the purpose of postprandial hyperglycemia mitigation during unannounced meals. Here, the results for the scenarios where the algorithms were first tuned and validated, and then a sensitivity analysis scenario where the algorithm is analyzed in depth to determine factors that affect detection performance are presented.

#### 3.2.1. Meal Detection Algorithm: Tuning

The tuning of the algorithm was done both by window size and threshold on the cross-covariance between $D_{diff}$ and the current CGM value. The window sizes and thresholds were empirically derived based on the sensitivity and the FP number, which were used as indicators of performance. In general, smaller window sizes and lower thresholds result in a higher sensitivity in the meal detection algorithm as seen in Table 2. The amount of carbohydrates consumed per meal by the 10 adult subjects in the tuning and validation scenarios had a mean of 47 ± 16 g and 47 ± 13 g and a median of 45 (23, 74) g and 46 (28, 69) g, respectively. These scenarios reflect carbohydrate consumption on a typical day for an average patient. Table 2 reports the mean, standard deviation, median, 5th percentile, and 95th percentile values for the sensitivity, Δ glucose, detection time, TP, FP, FN, and FP/day of each meal detection tuning. Typically, a higher sensitivity reflects lower Δ glucose, detection time, and FN values with increased TP, FP, and FP/day values. The high sensitivity tuning for both the tuning and validation scenarios had a mean sensitivity of 99 ± 1% and 99 ± 2% and a median of 99 (95, 100)% and 98 (90, 100)% and FP of mean 18 ± 6 and 20 ± 6 and median of 19 (9, 25) and 20 (11, 26). The trade-off tuning for both the tuning and validation scenarios had a mean sensitivity of 93 ± 5% and 94 ± 5% and median of 93 (86, 100)% and 94 (83, 100)% and FP of mean 4 ± 4 and 4 ± 3 and median 3 (0, 7) and 4 (1, 9). Finally, the low FP tuning for both the tuning and validation scenarios had a mean sensitivity of 47 ± 10% and 47 ± 16% and median of 50 (26, 64)% and 45 (29, 71)% and mean FP of 0 ± 0 and 0.2 ± 0.4 and median of 0 (0, 0) and 0 (0, 1).

#### 3.2.2. Meal Detection Algorithm: Sensitivity Analysis

**Figure 5:**
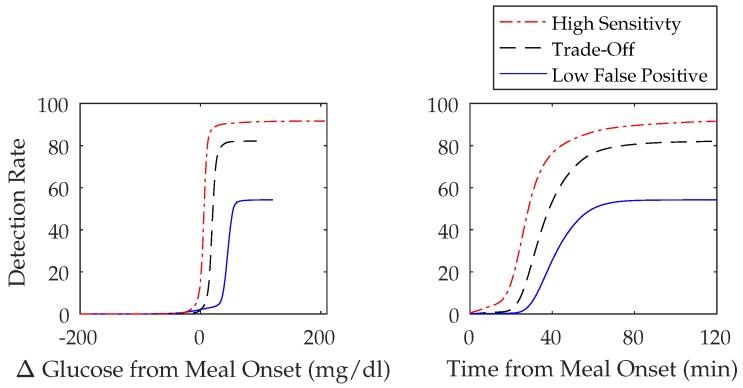
Cumulative detection rates over change in time and Δ glucose from the onset of meals for the meal algorithm tunings of high sensitivity, trade-off, and low false positive in the sensitivity analysis scenario.

A 500-day simulated scenario was used to perform a sensitivity analysis. The amount of carbohydrates consumed per meal by the 10 adult subjects had a mean of 70 ± 29 g and a median of 70 (25, 116) g. The population performance metrics can be found in Table 3, which, when compared to the three-meal algorithm tunings in the previous section (Section 3.2.1), shows a decrease in sensitivity for both the high sensitivity and trade-off tunings with mean values of 92 ± 3% and 82 ± 4% and median values of 92 (87, 96)% and 83 (76, 87)%, respectively. The low FP tuning, however, showed an increase in sensitivity with a mean value of 54 ± 9% and median value of 52 (44, 71)%. The FP/day between scenarios are roughly the same. The FP/day of the high sensitivity scenario for the tuning and validation scenarios and the sensitivity all shared a mean of 1 ± 1 and a median of 1 (0, 3). The FP/day in the trade-off scenario also had a similar value for the sensitivity analysis with a mean 0.2 ± 0.5 and median 0 (0, 1) versus mean 0.2 ± 0.5 and 0 ± 0 and median 0 (0, 1) and 0 (0, 0) of the tuning and validation scenarios. The FP/day in the low FP scenario was only slightly higher with a mean value of 0.02 ± 0.2 and median 0 (0, 0) versus mean 0 ± 0 and 0.01 ± 0.1 and median 0 (0, 0) and 0 (0, 0) in the tuning and validation scenarios. Figure 5 illustrates the cumulative detection rates over change in time and Δ glucose from the onset of meals for the three meal algorithm tunings and further emphasizes that algorithms with higher sensitivities have a shorter detection time and lower Δ glucose at detection.

An analysis of the effect of carbohydrate quantity (Table 4) on detection performance was performed. Carbohydrate quantities of 20–40 g have the longest detection time followed by 40–80 g and then 80–120 g. However, carbohydrate quantities of 20–40 g had the lowest Δ glucose at detection followed by 40–80 g and then 80–120 g. The meal detection sensitivity increased with increasing amounts of carbohydrates. The sensitivity for the highest sensitivity tuning was mean 74 ± 6% and median 72 (67, 87)% for 20–40 g of carbohydrates, mean 93 ± 3% and median 94 (87, 96)% for 40-80 g of carbohydrates, and mean 99 ± 1% and median 99 (97, 100)% for 80–120 g of carbohydrates. The sensitivity for the trade-off tuning was mean 49 ± 9% and median 47 (39, 64)% for 20–40 g of carbohydrates, mean 84 ± 4% and median 86 (76, 89)% for 40–80 g of carbohydrates, and mean 96 ± 2% and median 97 (94, 98)% for 80–120 g of carbohydrates. The sensitivity for the lowest FP tuning was mean 8 ± 7% and median 6 (2, 26)% for 20–40 g of carbohydrates, mean 49 ± 12% and median 45 (36, 71)% for 40–80 g of carbohydrates, and mean 83 ± 7% and median 82 (73, 93)% for 80–120 g of carbohydrates.

The effect of Ra on meal detection performance was also analyzed (Table 5). Meals with faster Ra values have a shorter detection time but a greater Δ glucose, while meals with slower Ra values take longer to detect but result in a lower Δ glucose before detection. The meal detection sensitivity increased with faster Ra values. The sensitivity for the highest sensitivity tuning was mean 77 ± 9% and median 77 (62, 92)% for meals with slow Ra values, mean 87 ± 4% and median 88 (81, 95)% for meals with medium Ra values, and mean 95 ± 1% and median 95 (92, 97)% for meals with fast Ra values. The sensitivity for the trade-off tuning was mean 54 ± 10% and median 57 (38, 71)% for meals with slow Ra values, mean 73 ± 6% and median 73 (65, 81)% for meals with medium Ra values, and mean 89 ± 3% and median 89 (84, 92)% for meals with fast Ra values. The sensitivity for the lowest FP tuning was mean 10 ± 8% and median 6 (2, 29)% for meals with slow Ra values, mean 33 ± 12% and median 29 (18, 57)% for meals with medium Ra values, and mean 68 ± 7% and median 67 (59, 81)% for meals with fast Ra values.

## 4. Discussion

An analysis of the states estimated by the UKF compared to the model states of the UVA/Padova Simulator was performed in Section 3.1. It was found that all states were estimated with reasonable precision. The states of $S_{1}$ and $S_{2}$ were found to have higher RMSE values, although both UKF estimated states seem to follow a similar trend to that of the UVA/Padova Simulator model states. The Ra between the UVA/Padova Simulator and the UKF was found to have a low RMSE of 2.8, however, the peak time and absorption dynamics appear to be quite different (see Figure 4). These differences must be taken into account if using this state to aid in bolus decisions.

In this study, three different tunings of the same meal detection algorithm to determine usefulness in warding off postprandial hyperglycemia were evaluated. This first tuning obtains the highest sensitivity possible as seen in Table 2 and Table 3 where sensitivities of mean 99 ± 2% and median 99 (95, 100)% for the tuning scenario, mean 98 ± 4% and median 98 (90, 100)% for the validation scenario, and mean 92 ± 3% and median 92 (87, 96)% for the sensitivity analysis scenario were achieved. The purpose of this high sensitivity tuning is to allow postprandial hyperglycemia mitigation action to be performed at the earliest possible instant. However, this tuning also contains a high FP value that increases risk of hypoglycemia due to insulin administration without meal ingestion.

The second tuning was a trade-off tuning that has both a high sensitivity with a low FP/day value. It obtained sensitivities of mean 93 ± 5% and median 93 (86, 100)% for the tuning scenario, mean 93 ± 6% and median 94 (83, 100)% for the validation scenario, and mean 82 ± 4% and median 83 (76, 87)% for the sensitivity analysis scenario (see Table 2 and Table 3). The FP/day values were mean 0.2 ± 0.5 and median 0 (0, 1) for the tuning scenario, mean 0 ± 0 and median 0 (0, 0) for the validation scenario, and mean 0.2 ± 0.5 and median 0 (0, 1) for the sensitivity analysis scenario. This tuning serves as a detection that is more reliable with less FP/day but has a longer detection time than that of the high sensitivity tuning, which entails a greater delay in insulin delivery and action.

The final tuning was a low FP tuning, originally the fixed FP value was evaluated during tuning but for the purposes of comparison between scenarios the FP/day values were used. The FP/day values were mean 0 ± 0 and median 0 (0, 0) for the tuning scenario, mean 0.01 ± 0.1 and median 0 (0, 0) for the validation scenario, and mean 0.02 ± 0.2 and median 0 (0, 0) for the sensitivity analysis scenario (see Table 2 and Table 3). This tuning allows postprandial hyperglycemia mitigation with very little risk of hypoglycemia due to FP detections. However, it has the longest detection time of the three tunings and its obtained sensitivities are quite low: mean 47 ± 12% and median 50 (26, 64)% for the tuning scenario, mean 46 ± 16% and median 45 (29, 71)% for the validation scenario, and mean 54 ± 9% and median 52 (44, 71)% for the sensitivity analysis scenario.

Several other meal detection algorithms have been created for use during unannounced meals and their comparable metrics can be found in Table 6. Dassau et al. [11] studied 17 patients using clinical data during a breakfast of 56 g with a range between 22 and 105 g. However, with only meal detection time, it is difficult to compare the performance between meal detection algorithms. Lee et al. [13] studied an in silico population of 100 patients during 72 h with an average meal size of 47.5 ± 25 g. Their meal detection algorithm can be compared to the trade-off tuning of this study when the sensitivity and detection time are compared. Although the sensitivities in this study in the tuning and validation scenarios are higher and comparable for the sensitivity analysis scenario, without an FP/day value, it is not possible to determine which meal detection algorithm outperforms the other. Chen et al. [16] studied 10,000 patients over three days. However, with only the sensitivity value, it is difficult to compare algorithms. Weimer et al. [17] studied 61 patients using clinical data over a period of 17 days. Their algorithm has both a lower sensitivity of 86.9% and high FP/day of 2.01.

Xie and Wang [18] studied 30 patients during two days with an average meal size of 61.7 ± 14.4 g. However, with only a sensitivity value, it is difficult to compare algorithms. Turksoy et al. [19] studied nine in clinic patients over 32 hours with an average meal size of 44 ± 9.4 g. This meal detection algorithm has both a high sensitivity of 97 ± 6% and low Δ glucose of 16 ± 9.4 mg/dL and, out of 63 meals and snacks, there was only one FP and two FN. Although their results outperform the results found in this study, the duration of this study was not long enough to truly reflect meal detection capabilities. Finally, Mahmoudi et al. [20] studied 10 in silico patients over 50 days. They achieved a high sensitivity of 99.5% however, their detection time was very long at 58.4 ± 18.7 min. In addition, additional information about FP or FP/day is not given, making it difficult to compare algorithms.

A sensitivity analysis was done to see the effect of carbohydrate quantity and meal composition on meal detection performance. Meal absorption related parameters of the UVA/Padova Simulator were varied in meals in such a way to suggest varying fat percentages, which ultimately affected Ra [32]. Although this is simulated data with a small cohort and meal variability limited to the meals included in the meal library, it gives insight into the effect of carbohydrate quantity and meal composition on detection capabilities. The population performance metrics (Table 3) differ from those in the tuning and validation scenario because of the increased amount of meal variability as mentioned in Section 2.5. In addition, meals in the sensitivity analysis scenario tended towards higher carbohydrate amounts due to the uniform distribution of meals between 20 and 120 g. It can been seen that there is a decrease in the sensitivities of the highest sensitivity and trade-off tunings with values of mean 92 ± 3% and median 92 (87, 96)% and mean 82 ± 4% and median 83 (76, 87)%, respectively. There is a slight increase in the sensitivity for the low FP tuning to mean 54 ± 9% and median 52 (44, 71)%. Interestingly, the number of FP/day remain very similar between scenarios.

An analysis of the effect of carbohydrate quantity (Table 4) on detection performance reveals that larger amounts of carbohydrates have a shorter detection time but a greater Δ glucose, while smaller quantities of carbohydrates take longer to detect but result in a lower glucose increase before detection due to lower amounts of carbohydrate ingestion. The meal detection sensitivity decreased with decreasing amounts of carbohydrates representing that small meals are generally more difficult to detect.

The effect of meal composition and ultimately Ra on meal detection performance was also analyzed (see Table 5). A similar trend was found when compared to carbohydrate quantity where just like large meals, meals with faster Ra values take less time to be detected but result in a greater Δ glucose. The meal detection sensitivity increased with faster Ra values representing that meals that are slowly digested or have a higher quantity of fat are generally more difficult to detect. This is consistent with the results reported by Bell et al. [33], which state that, during high fat meals, glucose values have a reduced area under the curve in the first 2–3 h due to a delayed gastric emptying rate.

This sensitivity analysis reveals that, fortunately, the meals of most interest, i.e., those that require both detection and postprandial hyperglycemia mitigation due to large disturbances in glucose, such as large and rapidly appearing meals, are able to be detected quickly with very little change in glucose at detection. However, meals with slow rates of glucose appearance such as those high in fat may be more prone to late-onset postprandial hyperglycemia.

## 5. Conclusions

Currently, hybrid closed-loop AP systems are gradually moving towards a fully closed-loop design. Therefore, the user of the system will be required to perform less actions and have less responsibility for their overall T1D management. The first step to closing the loop is removing meal announcement; however, many challenges still remain due to the insulin action lag time and the discrepancy in postprandial hyperglycemia, which is dependent on meal composition and intra-patient variability.

A meal detection algorithm that uses CGM values and a disturbance parameter estimated from an adapted minimal model using an UKF has been presented. Tuning of this meal detection algorithm is complicated by the need to administer insulin as soon as possible due to the lag in insulin action. Therefore, three tunings that can be useful for the mitigation of postprandial hyperglycemia have been implemented using the UVA/Padova simulator, an FDA accepted substitute for preclinical testing of novel technologies in diabetes care and a well-established starting point for evaluating the presented meal detection method.

Now that the algorithm and tunings have been established, future work will focus on using a combination of meal detection and feed-forward actions to test their ability to prevent postprandial hyperglycemia under conditions where meals have not been announced and there is a need for postprandial hyperglycemia mitigation strategies. Preliminary work will first be performed in silico and then, later on, these findings can be applied to a clinical setting for further testing.

## Figures and Tables

**Table 1:** Mean population parameter values.

Symbol	Quantity	Value	Units	Reference
$p_{1}$	Glucose removal rate from the plasma space independent of the influence of insulin	0.035	1/min	[27]
$p_{2}$	Disappearance rate of remote insulin from the remote insulin compartment	0.05	1/min	[27]
$p_{3}$	Appearance rate of remote insulin into the remote insulin compartment	0.000028	mL/μU · min${}^{2}$	[27]
$G_{b}$	Basal plasma glucose	100	mg/dL	[23]
$V_{G}$	Volume distribution of glucose compartment	1.6	dL/kg	[25]
$V_{I}$	Volume distribution of insulin compartment	120	mL/kg	[25]
$k_{e}$	First-order decay rate of insulin in plasma	0.138	1/min	[25]
$t_{max,I}$	Time-to-maximum insulin absorption	55	min	[25]
τ	Time constant of the system	8.2237	min	[24]
*g*	Static gain of the system	1	unitless	[24]

**Table 2:** Population performance metrics of meal detection algorithm in tuning scenario. Total number of meals per patient was 42.

	Sensitivity (%)	Δ Glucose (mg/dL)	Detection Time (min)	TP	FP	FN	FP/day
**Highest Sensitivity (window = 3; threshold = 0.000039)**							
**Tuning**	99 ± 2	7 ± 7	28 ± 10	41 ± 1	17 ± 5	1 ± 1	1 ± 1
	99 (95, 100)	6 (−1, 17)	25 (15, 45)	42 (40, 42)	19 (9, 25)	1 (0, 2)	1 (0, 3)
**Validation**	98 ± 4	6 ± 8	28 ± 9	41 ± 2	18 ± 5	1 ± 2	1 ± 1
	98 (90, 100)	5 (−2, 13)	25 (15, 40)	42 (38, 42)	20 (11, 26)	0 (0, 4)	1 (0, 3)
**Trade-Off (window = 9; threshold = 0.00019)**							
**Tuning**	93 ± 5	19 ± 5	37 ± 9	39 ± 2	3 ± 2	3 ± 2	0.2 ± 0.5
	93 (86, 100)	19 (11, 28)	35 (25, 55)	39 (36, 42)	3 (0, 7)	3 (0, 6)	0 (0, 1)
**Validation**	93 ± 6	19 ± 5	37 ± 83	39 ± 3	3 ± 3	3 ± 3	0 ± 0
	94 (83, 100)	18.43 (11, 26)	35 (25, 50)	40 (35, 42)	4 (1, 9)	3 (0, 7)	0 (0, 0)
**Lowest False Positive (window = 12; threshold = 0.00082)**							
**Tuning**	47 ± 12	45 ± 7	46 ± 8	20± 5	0 ± 0	22 ± 5	0 ± 0
	50 (26, 64)	45 (36, 54)	45 (35, 60)	21 (11, 27)	0 (0, 0)	21 (15, 31)	0 (0, 0)
**Validation**	46 ± 16	43 ± 7	48 ± 8	19 ± 7	0.2 ± 0.4	23 ± 7	0.01 ± 0.1
	45 (29, 71)	45 (35, 56)	50 (35, 60)	19 (12, 30)	0 (0, 1)	23 (12, 30)	0 (0, 0)

Values reported as mean ± standard deviation and median (5th%, 95th%). Abbreviations: TP, true positive; FP, false positive; FN, false negative.

**Table 3:** Population performance metrics of meal detection algorithm in sensitivity analysis scenario. Total number of meals per patient was 1500.

Sensitivity (%)	Δ Glucose (mg/dL)	Detection Time (min)	TP	FP	FN	FP/day
**Highest Sensitivity (window = 3; threshold = 0.000039)**						
92 ± 3	6 ± 13	31 ± 16	1375 ± 38	719 ± 111	126 ± 38	1 ± 1
92 (87, 96)	5 (−8, 17)	25 (10, 60)	1374 (1306, 1433)	716 (561, 897)	126 (67, 194)	1 (0, 3)
**Trade-Off (window = 9; threshold = 0.00019)**						
82 ± 4	19 ± 9	38 ± 14	1232 ± 56	100 ± 23	268 ± 56	0.2 ± 0.5
83 (76, 87)	19 (6, 31)	35 (25, 65)	1242 (1139, 1310)	96 (72, 154)	259 (190, 361)	0 (0, 1)
**Low False Positive (window = 12; threshold = 0.00082)**						
54 ± 9	42 ± 18	43 ± 11	813 ± 130	11 ± 9	687 ± 130	0.02 ± 0.2
52 (44, 71)	45 (10, 56)	40 (30, 65)	776 (663, 1060)	10 (5, 34)	724 (440, 837)	0 (0, 0)

Values reported as mean ± standard deviation and median (5th%, 95th%). Abbreviation: TP, true positive; FP, false positive; FN, false negative.

**Table 4:** Analysis of the effect of carbohydrate quantity on detection time, Δ glucose, and sensitivity.

			Carbohydrates (grams)		
			20–40	40–80	80–120
High Sensitivity window = 3 threshold = 0.000039	Detection Time (min)	Mean	39 ± 22	31 ± 16	25 ± 13
		Median	30 (10, 90)	30 (10, 60)	25 (0, 50)
	Δ Glucose (mg/dL)	Mean	5 ± 14	5 ± 10	7 ± 15
		Median	5 (−13, 23)	5 (−7, 16)	6 (−6, 15)
	Sensitivity (%)	Mean	74 ± 6	93 ± 3	99 ± 1
		Median	72 (67, 87)	94 (87,96)	99 (97, 100)
Trade-Off window = 9 threshold = 0.00019	Detection Time (min)	Mean	46 ± 18	40 ± 16	32± 15
		Median	45 (25, 80)	35 (25, 65)	30 (0, 55)
	Δ Glucose (mg/dL)	Mean	18 ± 11	17 ± 9	20 ± 9
		Median	19 (0, 32)	18 (0, 28)	20 (8, 32)
	Sensitivity (%)	Mean	49 ± 9	84 ± 4	96 ± 2
		Median	47 (39, 64)	86 (76, 89)	97 (94, 98)
Low False Positive window = 12 threshold = 0.00082	Detection Time (min)	Mean	45 ± 11	44 ± 8	41 ± 11
		Median	50 (14, 71)	45 (30, 65)	40 (30, 60)
	Δ Glucose (mg/dL)	Mean	29 ± 42	42 ± 18	42 ± 16
		Median	43 (−46, 57)	45 (12, 57)	44 (16, 56)
	Sensitivity (%)	Mean	8 ± 7	49 ± 12	83 ± 7
		Median	6 (2, 26)	45 (36, 71)	82 (73, 93)

Values reported as mean ± standard deviation and median (5th%, 95th%).

**Table 5:** Analysis of the effect of rate of glucose appearance on detection time, Δ glucose, and sensitivity.

			Rate of Glucose Appearance		
			Slow	Medium	Fast
High Sensitivity window = 3 threshold = 0.000039	Detection Time (min)	Mean	47 ± 22	38 ± 20	27 ± 11
		Median	45 (10, 90)	35 (10, 80)	25 (10, 45)
	Δ Glucose (mg/dL)	Mean	3 ± 15	4 ± 13	6 ± 13
		Median	4 (−22, 25)	4 (−14, 23)	6 (−5, 15)
	Sensitivity (%)	Mean	77 ± 9	87 ± 4	95 ± 1
		Median	77 (62, 92)	88 (81, 95)	95 (92, 97)
Trade-Off window = 9 threshold = 0.00019	Detection Time (min)	Mean	57 ± 18	47 ± 16	34 ± 10
		Median	55 (35, 90)	45 (30, 80)	30 (25, 50)
	Δ Glucose (mg/dL)	Mean	16 ± 14	18 ± 12	20 ± 7
		Median	18 (−5, 36)	19 (1, 33)	19 (9, 30)
	Sensitivity (%)	Mean	54 ± 10	73 ± 6	89 ± 3
		Median	57 (38, 71)	73 (65, 81)	89 (84, 92)
Low False Positive window = 12 threshold = 0.00082	Detection Time (min)	Mean	63 ± 14	52 ± 13	40 ± 9
		Median	65 (45, 80)	50 (35, 75)	40 (30, 55)
	Δ Glucose (mg/dL)	Mean	37 ± 44	43 ± 24	41 ± 15
		Median	48 (−39, 72)	47 (−2, 63)	44 (13, 55)
	Sensitivity (%)	Mean	10 ± 8	33 ± 12	68 ± 7
		Median	6 (2, 29)	29 (18, 57)	67 (59, 81)

Values reported as mean ± standard deviation and median (5th%, 95th%).

**Table 6:** Population performance metrics of meal detection algorithm in other studies.

Reference	Sensitivity (%)	Δ Glucose (mg/dL)	Detection Time (min)	TP	FP	FN	FP/day
Dassau et al. [11]	−	−	30	−	−	−	−
Lee et al. [13]	82	−	31	656	54	144	−
Chen et al. [16]	99.6	−	−	−	−	−	−
Weimer et al. [17]	86.9	−	−	−	−	−	2.01
Xie and Wang [18]	95	−	−	−	−	−	−
Turksoy et al. [19]	97 ± 6	16 ± 9	−	7 ± 2	0.1 ± 0.3	0.2 ± 0.4	−
Mahmoudi et al. [20]	99.5	46.3 ± 21.2	58.4 ± 18.7	−	−	−	−

Values reported as mean ± standard deviation. Abbreviation: TP, true positive; FP, false positive; FN, false negative.

## Abbreviations

The following abbreviations are used in this manuscript:

AP	Artificial Pancreas
BG	Blood Glucose
CGM	Continuous Glucose Monitor
FN	False Negative
FP	False Positive
HbA1c	Glycated Hemoglobin
Ra	Rate of Glucose Appearance
RMSE	Root Mean Squared Error
T1D	Type 1 Diabetes
TN	True Negative
TP	True Positive
UKF	Unscented Kalman Filter
UVA/Padova	University of Virginia/Padova
